# Efficacy and safety of ritlecitinib in Asian patients with alopecia areata: A subgroup analysis of the ALLEGRO phase 2b/3 trial

**DOI:** 10.1111/1346-8138.17539

**Published:** 2025-03-12

**Authors:** Xingqi Zhang, Yanting Ye, Weiling Sun, Youyu Sheng, Misaki Kinoshita‐Ise, Taisuke Ito, Cheng‐Che Lan, Ohsang Kwon, Gregor Schaefer, Robert Wolk, Shasha Hu, Qiankun Sun, Yimeng Shen, Masayo Sakaki‐Yumoto

**Affiliations:** ^1^ Department of Dermatology The First Affiliated Hospital, Sun Yat‐Sen University Guangzhou China; ^2^ The First Affiliated Hospital of Nanjing Medical University Nanjing Jiangsu China; ^3^ Huashan Hospital Fudan University Shanghai China; ^4^ Kyorin University Faculty of Medicine Tokyo Japan; ^5^ Hamamatsu University School of Medicine Hamamatsu Japan; ^6^ Department of Dermatology College of Medicine and Chung‐Ho Memorial Hospital, Kaohsiung Medical University Kaohsiung Taiwan; ^7^ Seoul National University College of Medicine Seoul South Korea; ^8^ Pfizer Pharma GmbH Berlin Germany; ^9^ Pfizer Inc Groton Connecticut USA; ^10^ Pfizer China R&D Beijing China; ^11^ Pfizer China R&D Shanghai China; ^12^ Pfizer PBG Beijing China; ^13^ Pfizer Japan Inc Tokyo Japan

**Keywords:** ALLEGRO, alopecia areata, ritlecitinib, SALT, Severity of Alopecia Tool

## Abstract

This subgroup analysis of the ALLEGRO phase 2b/3 study (NCT3732807) assessed the efficacy and safety of multiple doses of ritlecitinib, an oral JAK3/TEC family kinase inhibitor, in Asian patients with alopecia areata (AA). Patients aged ≥12 years with AA and ≥50% scalp hair loss received once‐daily ritlecitinib 50 or 30 mg (with or without 4‐week 200‐mg loading dose [“200/50” or “200/30”]) or 10 mg or placebo for 24 weeks, followed by a 24‐week extension, in which patients initially assigned to placebo switched to 200/50 or 50 mg. In this subgroup analysis, Asian patients with response based on achieving a Severity of Alopecia Tool (SALT) score ≤20, SALT ≤10, ≥2‐grade improvement or normal score on the eyebrow assessment (EBA) scale, and ≥2‐grade improvement or normal score on the eyelash assessment (ELA) scale were evaluated through week 48. Safety was monitored throughout. In total, 186 Asian patients were randomized to ritlecitinib 200/50 mg (*n* = 33), 200/30 mg (*n* = 28), 50 mg (*n* = 43), 30 mg (*n* = 34), 10 mg (*n* = 17), placebo to 200/50 mg (*n* = 14), or placebo to 50 mg (*n* = 17). The proportions of patients treated with ritlecitinib ≥30 mg achieving a SALT score ≤20 response were 9.1%–36.4% at week 24 vs 0% for the 10‐mg group and 3.2% for placebo. At week 48, 26.5%–55.6% of patients treated with ritlecitinib ≥30 mg achieved a SALT ≤20 response. At week 48, the proportions of patients treated with ritlecitinib ≥30 mg with EBA response were 41.9%–71.1% and with ELA response were 40.7%–57.9%. The most common adverse events were nasopharyngitis, folliculitis, upper respiratory tract infection, and urticaria. No serious or opportunistic infections, major adverse cardiovascular events, thromboembolic events, malignancies, or deaths were reported. Ritlecitinib demonstrated clinical efficacy and acceptable safety over 48 weeks in Asian patients ≥12 years with AA and ≥50% hair loss. Results for the Asian subpopulation were consistent with the overall population in the ALLEGRO‐2b/3 study.

## 1 INTRODUCTION

Alopecia areata (AA) is an autoimmune disorder with an underlying immuno‐inflammatory pathogenesis, caused by inflammation in the lower region of the hair follicle leading to damage to the hair follicle.^1^, ^2^, ^3^, ^4^ Interferon gamma (IFN‐γ) is one of the key factors involved in the collapse of hair follicle immune privilege.^5^ This in turn leads to an aggregation of immune cells around the lower hair follicle, including CD4^+^ (T‐helper) cells, CD8^+^ cells, and natural killer (NK) cells.^5^, ^6^, ^7^, ^8^ This attack on the hair follicle ultimately leads to nonscarring hair loss. This can present as patchy scalp hair loss, complete loss of scalp hair (alopecia totalis [AT]), or total loss of hair on the scalp, face (eyebrows, eyelashes, beard, etc.), and body (alopecia universalis [AU]). AA can affect adults and children across all ages, races, and sexes.^9^, ^10^, ^11^

Many patients with AA, of all ages, experience psychological health issues, including an increased incidence of anxiety and depressive disorders.^12^ Other psychological issues include reduced self‐esteem, impaired sleep quality, and problems concerning social relations.^9^, ^13^, ^14^, ^15^, ^16^, ^17^, ^18^

AA is estimated to affect approximately 2% of the global population^19^; however, data on the prevalence of AA in countries across Asia are limited. One study conducted between 2007 and 2008 across six provinces in China estimated the prevalence of AA to be 0.24%,^20^ while studies in Japan have reported prevalence estimates of 1.45%–2.5%.^21^, ^22^ While AA can occur in people of all races, studies investigating whether AA disproportionately occurs in certain subgroups have shown contradictory results.^1^, ^10^, ^11^, ^23^ One study conducted in the UK found a threefold higher incidence of AA in people of Asian origin compared with those who were White.^24^

Patients with AA, especially severe AA, may require long‐term systemic therapy. However, there has historically been a lack of long‐term effective and safe systemic treatments indicated for AA. Treatments for AA have been historically mostly administered off‐label and include topical corticosteroids, intralesional corticosteroid injections, systemic corticosteroids, oral corticosteroids, and contact immunotherapy.^8^, ^25^, ^26^, ^27^, ^28^ However, many of these treatments show variable clinical responses. Furthermore, oral corticosteroids are associated with significant adverse events (AEs) such as decreased bone density, infections, edema, changes in blood pressure, and decreased bone growth among adolescents (among others). As such, oral corticosteroids are not suitable for long‐term use.^8^, ^29^, ^30^, ^31^, ^32^, ^33^, ^34^ Recently, the Janus kinase (JAK) 1/2 inhibitor baricitinib was approved in a number of countries, including the USA, Europe, Japan, and China, for the treatment of adults with severe AA.^35^

Ritlecitinib is a highly selective oral JAK3 inhibitor that also selectively inhibits all five members of the tyrosine kinase expressed in the hepatocellular carcinoma (TEC) kinase family (Bruton tyrosine kinase [BTK], bone marrow kinase on the X chromosome [BMX], interleukin[IL]‐2 inducible T‐cell kinase [ITK], tyrosine kinase expressed in T cells [TXK], and TEC).^36^, ^37^, ^38^ Via inhibition of JAK3, ritlecitinib was shown to inhibit the signaling of the γ‐chain inflammatory cytokines, including interleukin (IL)‐2, IL‐4, IL‐7, IL‐15, and IL‐21.^39^ A reduction in IL‐2 signaling reduces the downstream signaling of the T‐cell receptor, thus preventing the generation of autoreactive CD8^+^ T cells that are central to the pathogenesis of AA.^40^, ^41^, ^42^, ^43^ At the same time, ritlecitinib inhibition of TEC family kinases results in inhibition of CD8^+^ T cell and NK cell cytolytic activity as measured by reductions in CD107 and IFN‐γ, and inhibits antigen receptor signaling in B cells as measured by CD69 expression.^37^ In a biopsy substudy conducted during the 24‐week placebo‐controlled period of the ALLEGRO phase 2a clinical trial, ritlecitinib suppressed inflammatory T cells (by significantly decreasing CD3^+^ and CD8^+^ T‐cell counts) and reduced expression of inflammatory biomarkers such as IL‐9, IFN‐γ, CCL18, and CXCL10.^39^

Ritlecitinib was evaluated in the ALLEGRO phase 2b/3 study (NCT03732807), in which it demonstrated efficacy and an acceptable safety profile in patients aged ≥12 years with AA with ≥50% scalp hair loss.^44^ These positive results from the ALLEGRO study led to the approval of ritlecitinib 50 mg in 2023 in the USA and Europe for the treatment of adolescents and adults with severe AA. In the same year, ritlecitinib 50 mg was approved in Japan for the treatment of AA (limited to intractable cases involving widespread hair loss) and it is also the first new drug approved in China for adolescents and adults with severe AA. This subgroup analysis specifically evaluated the efficacy and safety of ritlecitinib in Asian patients from the ALLEGRO‐2b/3 study.

## 2 METHODS

### 2.1 Study design

The design and primary results of the ALLEGRO‐2b/3 study were previously reported.^44^ ALLEGRO phase 2b/3 was an international, randomized, double‐blind, placebo‐controlled, combined dose‐ranging and pivotal trial. Patients were randomized (2:2:2:2:1:1:1) to one of the following ritlecitinib treatment regimens: 50 or 30 mg (with or without a 200‐mg once‐daily [QD] loading dose for the initial 4 weeks, referred to as “200/50” or “200/30”), or 10 mg, or one of two placebo groups for 24 weeks (Figure S1). The 10‐mg group was assessed for pharmacokinetic and dose‐ranging purposes only. During a subsequent 24‐week extension period, ritlecitinib groups continued with their initial dose (50‐, 30‐, or 10‐mg) and patients who were initially randomized to placebo during the dose‐ranging period switched to ritlecitinib 50 mg QD with or without a 200‐mg, 4‐week loading dose.

### 2.2 Patient population

The study included patients ≥12 years of age with a clinical diagnosis of AA with ≥50% scalp hair loss, with no other etiology of hair loss, a current episode of hair loss ≤10 years, and no terminal hair regrowth within the previous 6 months. Exclusion criteria included other causes of alopecia, clinically significant depression, previous use of any JAK inhibitor, history of disseminated herpes zoster (HZ) or disseminated herpes simplex, or recurrent localized dermatomal HZ. This subgroup analysis included self‐identified Asian patients, defined as those of Asian descent in the overall ALLEGRO‐2b/3 study population. Written informed consent was obtained from each patient, parent, or patient's legal representative.

### 2.3 Outcomes

The primary endpoint of the overall study was response based on absolute Severity of Alopecia Tool (SALT) score ≤20 (≤20% scalp hair loss) at week 24. SALT assesses the amount of scalp hair loss with scores ranging from 0 (no scalp hair loss) to 100 (complete scalp hair loss). Clinician‐reported outcomes included:

- proportion of patients with response based on an absolute SALT score ≤20 (≤20% of scalp without hair) through week 48
- proportion of patients with response based on an absolute SALT score ≤10 (≤10% of scalp without hair) through week 48
- change from baseline in absolute SALT score through week 48
- proportion of patients with ≥2‐grade improvement from baseline or a normal score on the eyebrow assessment (EBA) scale through week 48 among patients without normal EBA at baseline
- proportion of patients with ≥2‐grade improvement from baseline or a normal score on the eyelash assessment (ELA) scale through week 48 among patients without normal ELA at baseline.

EBA and ELA are 4‐point scales ranging from 0 (none, or no eyebrows/eyelashes) to 3 (normal eyebrows/eyelashes).

Patient‐reported outcomes included the proportion of patients with Patient Global Impression of Change (PGI‐C) response of “moderately improved” or “greatly improved” through week 48. PGI‐C is a self‐reported, single‐item scale in which patients rate the improvement or worsening of AA compared with the start of the study, using a scale of seven responses ranging from “greatly improved” to “greatly worsened.”

To assess safety, the occurrence of AEs was monitored throughout the study. AEs of interest (including opportunistic infections and cardiovascular, malignancy, neurological, and audiological events) were reviewed by independent external adjudication committees using predefined criteria. Clinical laboratory parameters, vital signs, electrocardiograms, physical findings, and other observations related to safety were also monitored throughout the study.

### 2.4 Statistical analysis

Descriptive analyses were used to summarize efficacy outcomes in Asian patients. For binary endpoints, the number of responders, percentage, and 95% confidence interval (CI) are presented. The CIs were calculated based on normal approximation. For continuous endpoints, a mixed‐effect model with repeated measures was used. This model included the factors (fixed effects) for treatment group, visit, treatment‐by‐visit interaction, and relevant baseline value when modeling the change from baseline. Estimates of least squares (LS) mean values and the 95% CIs at each visit for each treatment group were derived from the model.

Placebo groups were combined at week 24 for analyses and referred to as the pooled placebo group. Patients with missing SALT scores due to COVID‐19‐related reasons were excluded, and patients with missing SALT scores due to other reasons were considered nonresponders. An analysis was conducted in which all patients with missing data (EBA, ELA, and/or PGI‐C) were considered as nonresponders regardless of the reason.

## 3 RESULTS

### 3.1 Patients

Of the total 718 patients randomized, 186 (25.9%) were of Asian descent and were included in this analysis. Of these, 166 (89.2%) patients completed the study and 20 (10.8%) discontinued; reasons for patient discontinuation from the study are shown in Supporting Information Table S1. In total, 146 patients were from East Asian countries or regions including China (*n* = 81), Japan (*n* = 47), Taiwan (*n* = 11), and Korea (*n* = 7).

Baseline characteristics were generally similar across treatment groups (Table 1). Of the total Asian population (*n* = 186), mean (standard deviation [SD]) age was 30.6 (11.2) years, 76.9% were adults aged 18–44 years, and 10.8% were adolescents aged 12–17 years. Overall, 60.2% of patients were female. A total of 94 (50.5%) patients were classified as having AT/AU (defined as a SALT score of 100) and mean (SD) baseline SALT score ranged from 88.9 (15.1) to 93.4 (12.0) across treatment groups. No patients discontinued from the study due to COVID‐19‐related AEs.

**TABLE 1:** Baseline demographics and disease characteristics in Asian patients.

	Placebo ➔ ritlecitinib 50 mg (*n* = 17)	Placebo ➔ ritlecitinib 200/50 mg (*n* = 14)	Ritlecitinib 10 mg (*n* = 17)	Ritlecitinib 30 mg (*n* = 34)	Ritlecitinib 50 mg (*n* = 43)	Ritlecitinib 200/30 mg (*n* = 28)	Ritlecitinib 200/50 mg (*n* = 33)
Age							
Mean (SD), years	29.8 (7.2)	31.3 (8.7)	33.4 (12.2)	30.0 (10.2)	31.6 (11.9)	29.0 (11.9)	30.0 (13.0)
12–17 years, *n* (%)	0	0	2 (11.8)	3 (8.8)	3 (7.0)	7 (25.0)	5 (15.2)
≥18 years, *n* (%)	17 (100.0)	14 (100.0)	15 (88.2)	31 (91.2)	40 (93.0)	21 (75.0)	28 (84.8)
≥65 years, *n* (%)	0	0	0	0	1 (2.3)	0	1 (3.0)
Sex, *n* (%)							
Female	11 (64.7)	8 (57.1)	13 (76.5)	17 (50.0)	21 (48.8)	19 (67.9)	23 (69.7)
Disease duration since diagnosis, years							
Mean (SD)	8.3 (6.9)	9.5 (7.5)	6.1 (3.7)	6.4 (5.7)	7.2 (7.5)	7.0 (7.4)	6.7 (4.5)
Median (range)	6.7 (2.0, 26.5)	8.8 (0.4, 23.5)	4.9 (0.8, 13.6)	4.9 (0.0, 24.7)	6.0 (0.5, 44.7)	4.8 (0.0, 34.1)	6.2 (0.3, 17.0)
Duration of onset of the current episode, years							
Mean (SD)	3.4 (3.0)	3.3 (2.5)	4.1 (2.5)	3.9 (2.6)	3.6 (2.8)	2.8 (2.4)	3.6 (3.0)
Median (range)	2.8 (0.3, 9.7)	2.5 (0.4, 8.1)	3.9 (0.7, 9.7)	3.3 (0.5, 9.9)	3.1 (0.2, 9.0)	2.4 (0.0, 8.7)	2.8 (0.3, 9.6)
Type of current episode of AA, *n* (%)							
AT/AU^a^	8 (47.1)	7 (50.0)	10 (58.8)	21 (61.8)	18 (41.9)	14 (50.0)	16 (48.5)
AT	2 (11.8)	5 (35.7)	5 (29.4)	9 (26.5)	5 (11.6)	9 (32.1)	3 (9.1)
AU	5 (29.4)	2 (14.3)	2 (11.8)	7 (20.6)	7 (16.3)	2 (7.1)	7 (21.2)
Not specified^b^	1 (5.9)	0	3 (17.6)	5 (14.7)	6 (14.0)	3 (10.7)	6 (18.2)
Non‐AT/non‐AU^c^	9 (52.9)	7 (50.0)	7 (41.2)	13 (38.2)	25 (58.1)	14 (50.0)	17 (51.5)

Abbreviations: AA, alopecia areata; AT, alopecia totalis; AU, alopecia universalis; CRF, case report form; SALT, Severity of Alopecia Tool; SD, standard deviation.

^a^ Patients in the AT/AU category had a SALT score of 100% at baseline (regardless of the category in the AA history CRF). AT and AU subcategories were based on the AA history CRF.

^b^ Patients with a SALT score of 100% at baseline who were not classified as AT or AU in the AA history CRF.

^c^ Non‐AT/non‐AU, SALT score of <100% at baseline (regardless of the category in the AA history CRF).

### 3.2 Efficacy

**FIGURE 1:**
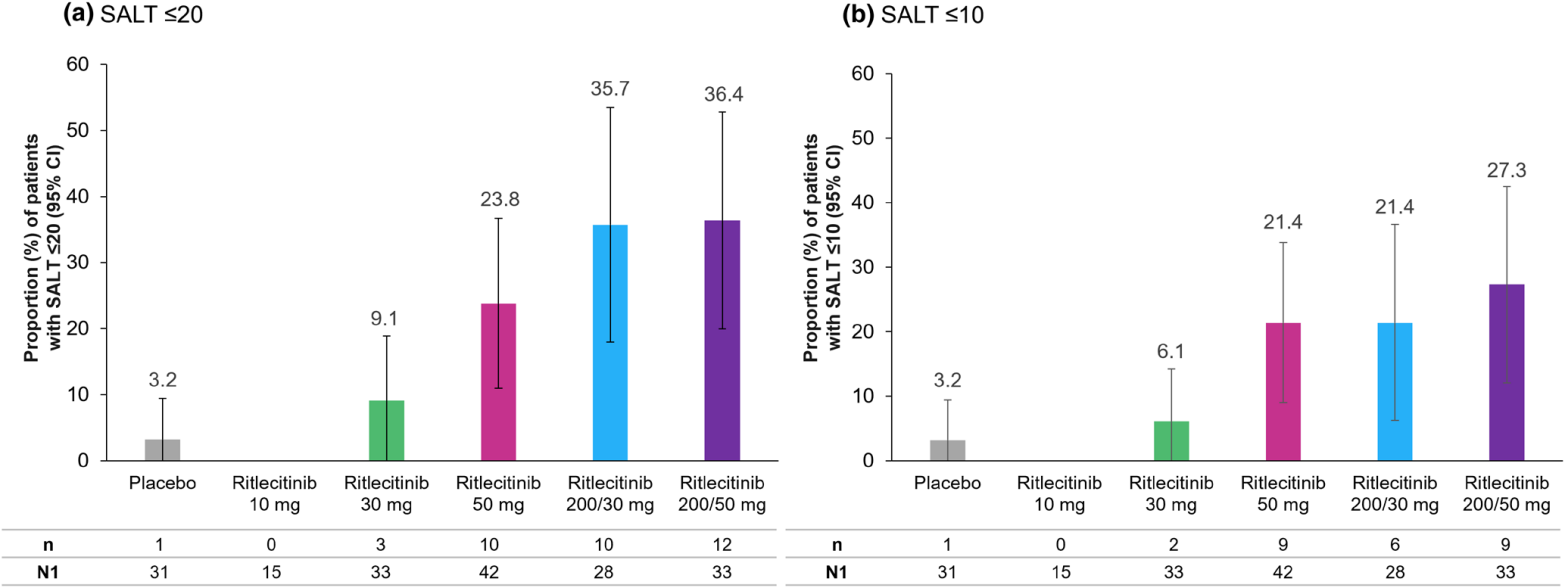
Proportion of Asian patients from the ALLEGRO‐2b/3 study with response based on (a) Severity of Alopecia Tool (SALT) score ≤20 and (b) SALT score ≤10 at week 24. CI, confidence interval; n, number of patients with SALT score ≤20 or ≤10 response; N1, number of patients with valid data at week 24 (nonresponse for missing due to reasons unrelated to COVID‐19, excludes missing due to COVID‐19). Percentage based on N1.

**FIGURE 2:**
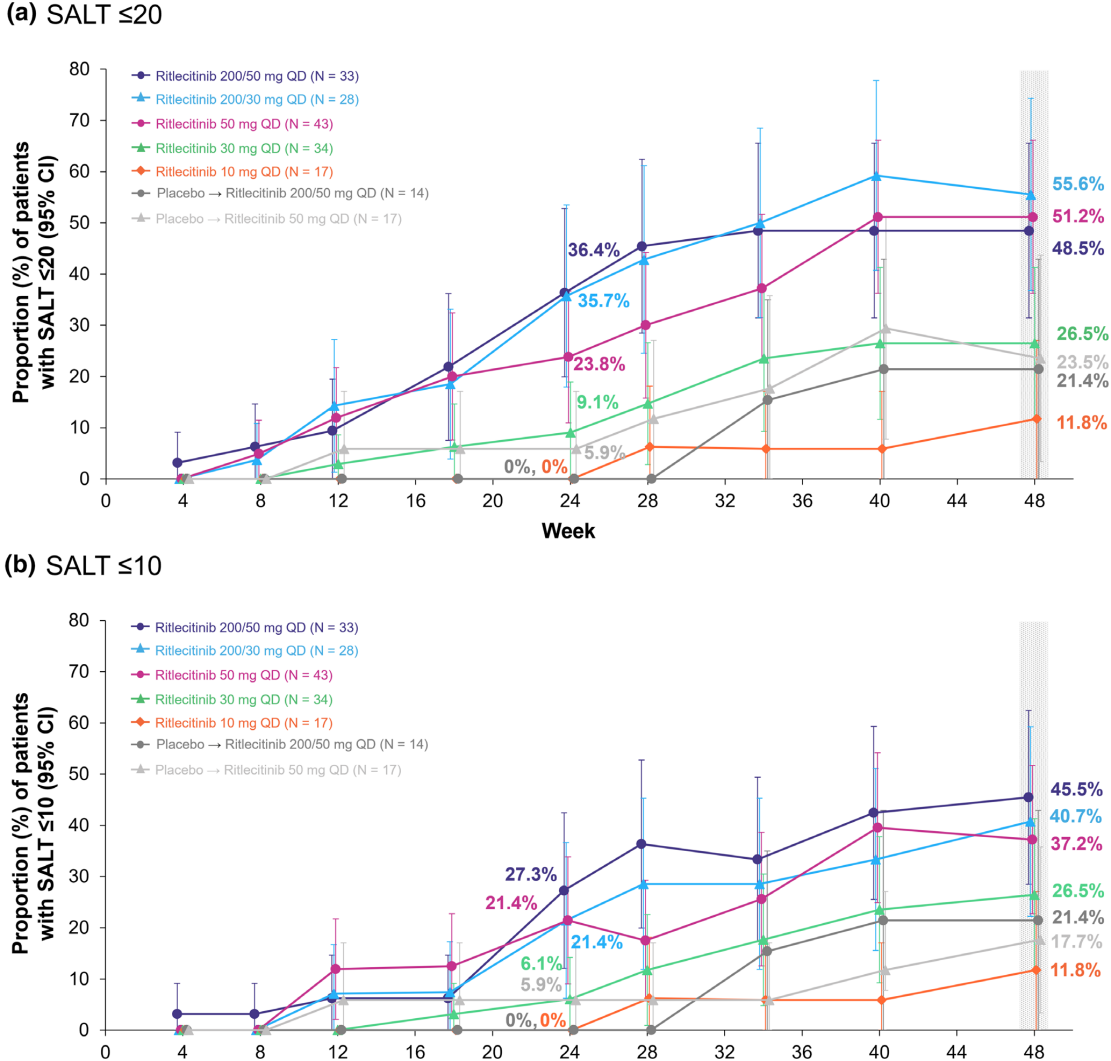
Proportion of Asian patients from the ALLEGRO‐2b/3 study with response based on (a) Severity of Alopecia Tool (SALT) score ≤20 and (b) SALT score ≤10 over time to week 48. CI, confidence interval; QD, once daily.

The primary endpoints of the ALLEGRO‐2b/3 study in the overall population were met.^44^ In this subgroup analysis of Asian patients, higher proportions of patients treated with ritlecitinib ≥30 mg achieved SALT score ≤20 response at week 24 (36.4%, 35.7%, 23.8%, and 9.1% in the 200/50‐, 200/30‐, 50‐, and 30‐mg groups, respectively) versus placebo (3.2%) (Figure 1a). Higher proportions of patients receiving ritlecitinib ≥30 mg also achieved response with the more stringent endpoint of SALT score ≤10 at week 24 (27.3%, 21.4%, 21.4%, and 6.1% in the 200/50‐, 200/30‐, 50‐, and 30‐mg groups, respectively) versus placebo (3.2%) (Figure 1b). At week 48, the proportions of patients with SALT ≤20 response were 48.5%, 55.6%, 51.2%, and 26.5% in the 200/50‐, 200/30‐, 50‐, and 30‐mg groups, respectively (Figure 2a), and with SALT ≤10 response were 45.5%, 40.7%, 37.2%, and 26.5%, respectively (Figure 2b). Among patients who switched from placebo to ritlecitinib 200/50 or 50 mg at week 24, 21.4% and 23.5% had a SALT score ≤20, respectively (Figure 2a), and 21.4% and 17.7% had a SALT score ≤10, respectively, at week 48 (Figure 2b).

**FIGURE 3:**
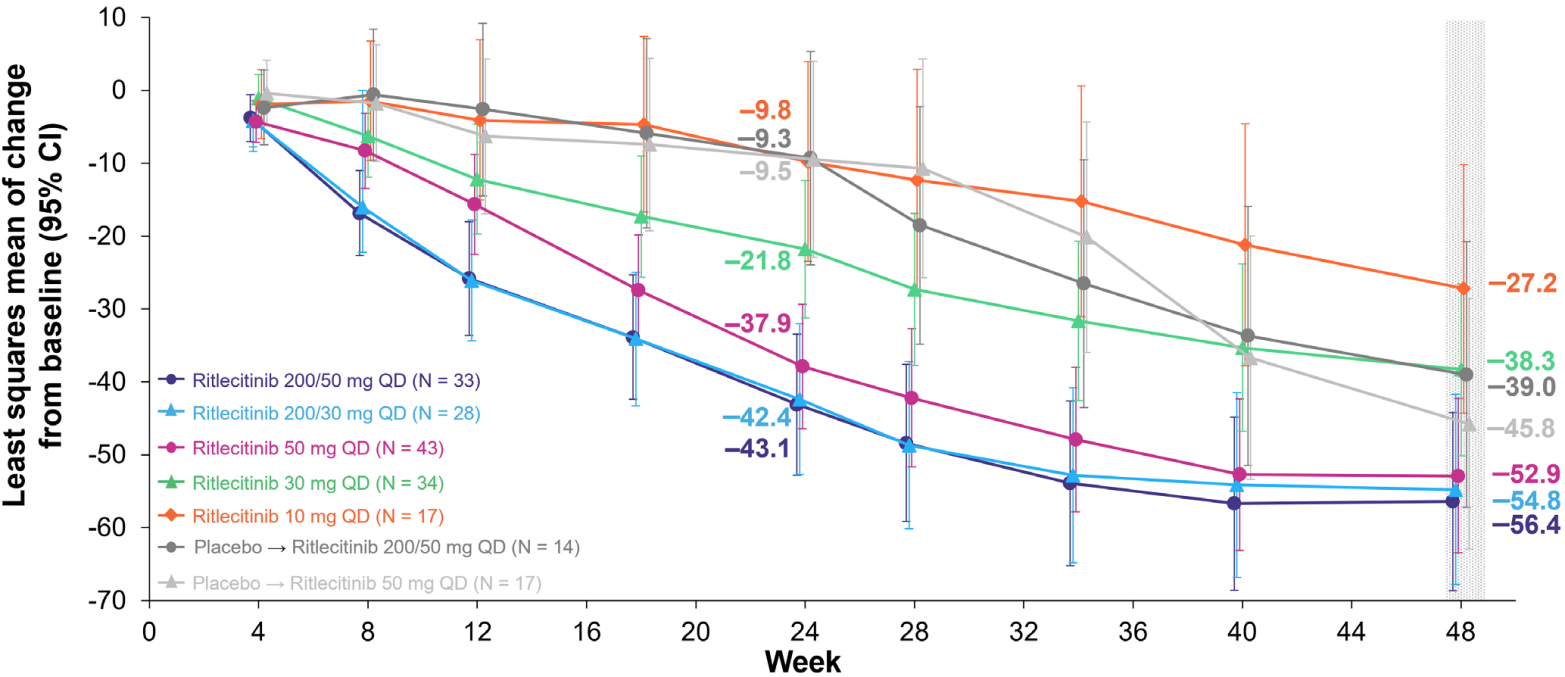
Change from baseline in Severity of Alopecia Tool score over time to week 48 in Asian patients from the ALLEGRO‐2b/3 study. CI, confidence interval; QD, once daily.

LS mean change from baseline in SALT score demonstrated a dose‐dependent effect: at week 48, estimated LS mean change in SALT score from baseline was −56.4, −54.8, −52.9, −38.3, and −27.2 for the 200/50‐, 200/30‐, 50‐, 30‐, and 10‐mg groups, respectively (Figure 3). Among patients who switched from placebo to ritlecitinib 200/50 or 50 mg at week 24, the mean change in SALT score from baseline at week 48 was −39.0 and −45.8, respectively.

**FIGURE 4:**
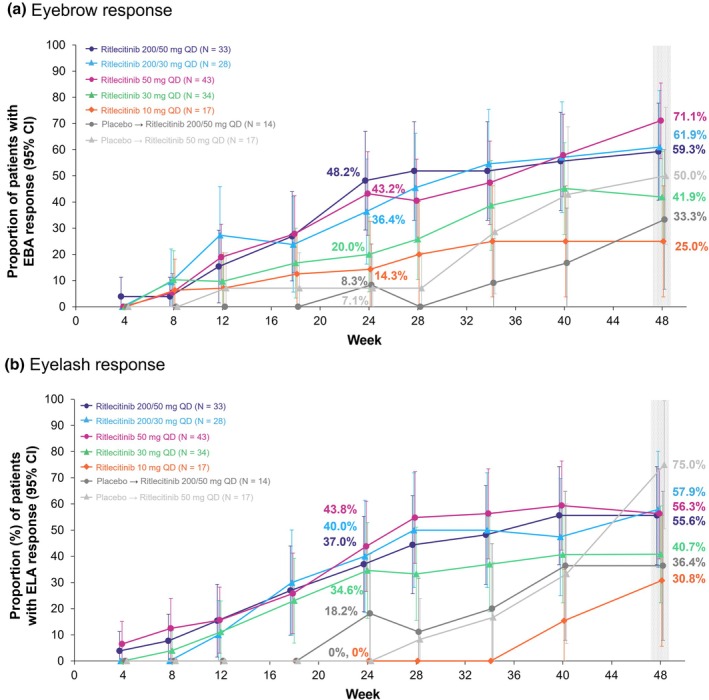
Proportions of Asian patients from the ALLEGRO‐2b/3 study with (a) eyebrow response (≥2‐grade improvement or normal eyebrow assessment [EBA] score) and (b) eyelash response (≥2‐grade improvement or normal eyelash assessment [ELA] score) from baseline to week 48. CI, confidence interval; QD, once daily.

Among patients without normal EBA at baseline, the proportions of patients who achieved ≥2‐grade improvement from baseline at week 24 were 48.2%, 36.4%, 43.2%, and 20.0% for the ritlecitinib 200/50‐, 200/30‐, 50‐, and 30‐mg groups, respectively, versus 8.3% and 7.1% in the placebo groups (Figure 4a). The proportion of patients with EBA response increased between weeks 24 and 48 in the 200/50‐, 200/30‐, 50‐, and 30‐mg groups; at week 48, 59.3%, 61.9%, 71.1%, and 41.9% of patients achieved EBA response. Among patients who switched from placebo to ritlecitinib 200/50 or 50 mg at week 24, 33.3% and 50.0% achieved EBA response, respectively, at week 48.

Among patients without normal ELA at baseline, the proportions of patients who achieved ≥2‐grade improvement from baseline at week 24 were 37.0%, 40.0%, 43.8%, and 34.6% for the ritlecitinib 200/50‐, 200/30‐, 50‐, and 30‐mg groups, respectively, versus 18.2% and 0% in the placebo groups (Figure 4b). An increase was seen in the proportion of patients with ELA response from week 24 in the ritlecitinib 200/50‐, 200/30‐, 50‐, and 30‐mg groups; at week 48, the proportions of patients with ELA response were 55.6%, 57.9%, 56.3%, and 40.7%, respectively. Among patients who switched from placebo to ritlecitinib 200/50 or 50 mg at week 24, 36.4% and 75.0% achieved ELA response, respectively, at week 48.

**FIGURE 5:**
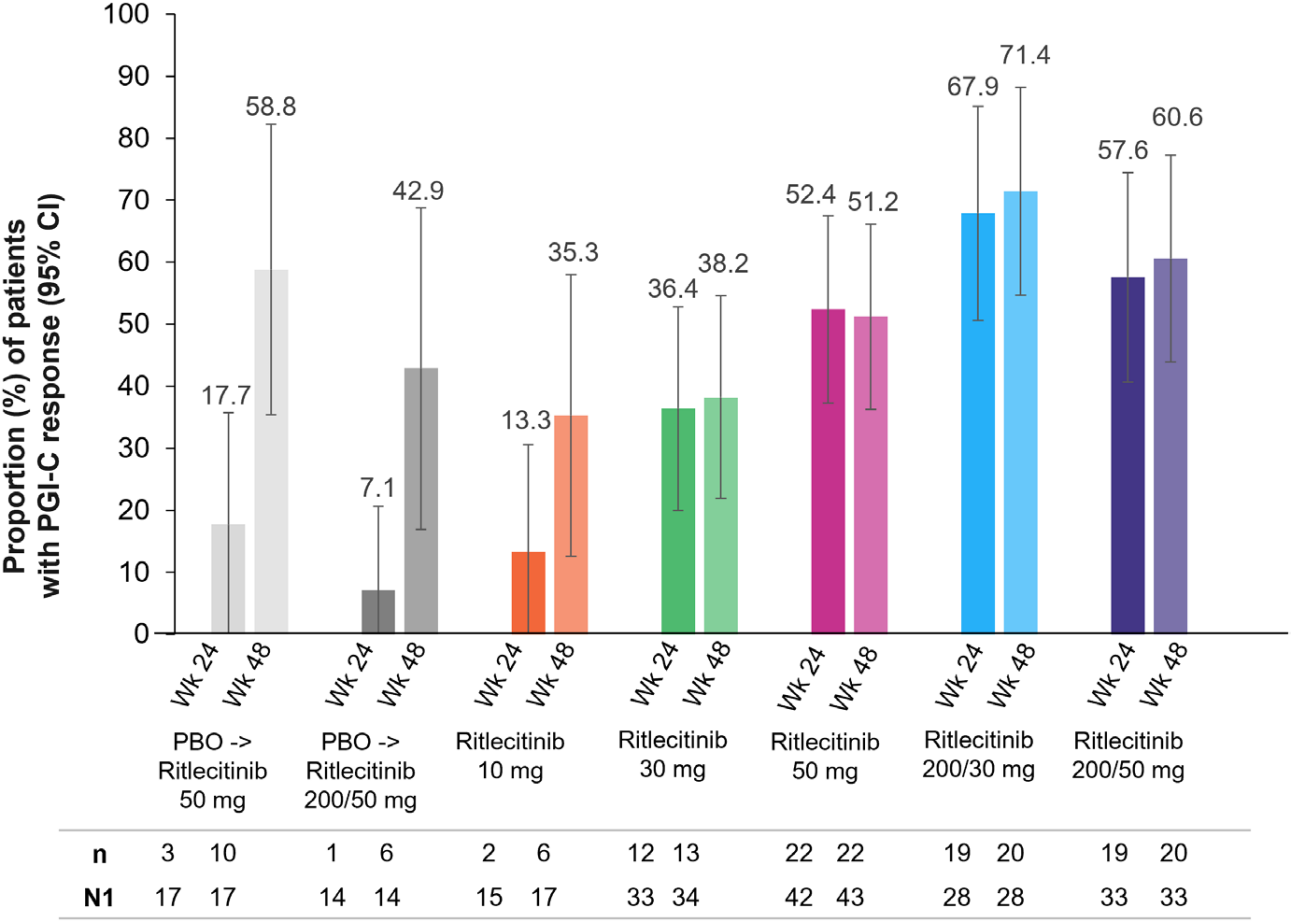
Proportions of Asian patients from the ALLEGRO‐2b/3 study with Patient Global Impression of Change (PGI‐C) response^a^ at weeks 24 and 48. ^a^PGI‐C response is defined as “moderately improved” or “greatly improved.” CI, confidence interval; N, number of patients with PGI‐C response; N1, number of patients with valid data at the analysis visit (nonresponse for missing due to reasons unrelated to COVID‐19, excludes missing due to COVID‐19); PBO, placebo. Percentage based on N1.

At week 24, the proportions of patients who achieved a PGI‐C response were 57.6%, 67.9%, 52.4%, and 36.4% in the ritlecitinib 200/50‐, 200/30‐, 50‐, and 30‐mg treatment groups, respectively, versus 12.9% in the pooled placebo groups; at week 48, the proportions of patients who achieved a PGI‐C response were 60.6%, 71.4%, 51.2%, and 38.2% in the respective ritlecitinib treatment groups (Figure 5). Among patients who switched from placebo to ritlecitinib 200/50 or 50 mg at week 24, 42.9% and 58.8% achieved a PGI‐C response, respectively, at week 48.

**FIGURE 6:**
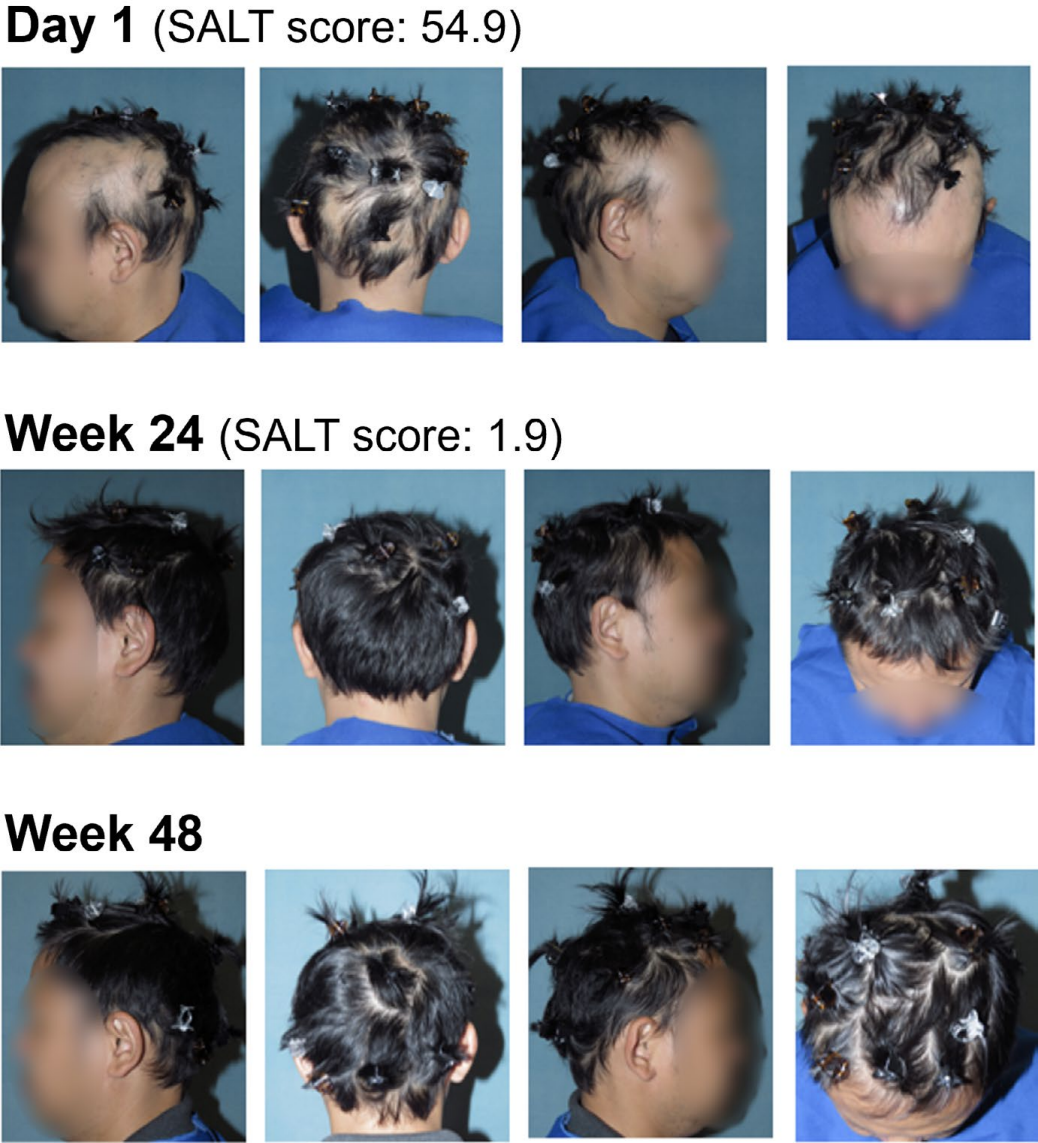
Representative photographs of an Asian patient from the subgroup analysis of the ALLEGRO‐2b/3 study with hair regrowth response to ritlecitinib 50 mg. SALT, Severity of Alopecia Tool.

Representative photographs of an Asian patient who responded to ritlecitinib treatment are shown in Figure 6.

### 3.3 Safety

During the placebo‐controlled period (weeks 0–24), AEs were reported in 64.7%–85.7% of patients across the ritlecitinib groups and in 67.7% of patients in the placebo group (Table 2). The most common AEs included nasopharyngitis, folliculitis, upper respiratory tract infection, and urticaria; most events were mild to moderate in severity. Throughout the entire study (up to week 48), AEs were reported in 70.6%–89.3% of patients in the ritlecitinib groups, and in 92.9% and 82.4% of patients in the placebo to ritlecitinib 200/50‐mg and placebo to 50‐mg groups, respectively (Table 2).

**TABLE 2:** Summary of AEs, SAEs, severe AEs, and discontinuations due to AEs with once‐daily ritlecitinib or placebo in Asian patients.

Patients with AE, *n* (%)	Placebo (pooled) (*n* = 31)	Ritlecitinib 10 mg (*n* = 17)	Ritlecitinib 30 mg (*n* = 34)	Ritlecitinib 50 mg (*n* = 43)	Ritlecitinib 200/30 mg (*n* = 28)		Ritlecitinib 200/50 mg (*n* = 33)
Placebo‐controlled period (up to week 24)							
Patients with AEs	21 (67.7)	11 (64.7)	25 (73.5)	33 (76.7)	24 (85.7)		27 (81.8)
Most common AEs occurring in ≥15% of patients in any treatment group							
Nasopharyngitis	2 (6.5)	3 (17.6)	3 (8.8)	4 (9.3)	8 (28.6)		3 (9.1)
Folliculitis	0	2 (11.8)	2 (5.9)	2 (4.7)	6 (21.4)		8 (24.2)
Upper respiratory tract infection	3 (9.7)	0	3 (8.8)	4 (9.3)	2 (7.1)		8 (24.2)
Urticaria	2 (6.5)	0	3 (8.8)	5 (11.6)	2 (7.1)		5 (15.2)
Permanent discontinuation from study or study drug due to AEs	2 (6.5)	1 (5.9)	1 (2.9)	2 (4.7)	0		2 (6.1)
Patients with SAEs	1 (3.2)	0	0	0	0		1 (3.0)
Patients with severe AEs	1 (3.2)	1 (5.9)	0	0	0		0
	**Placebo ➔ ritlecitinib 50 mg (** ***n*** ** = 17)**	**Placebo ➔ ritlecitinib 200/50 mg (** ***n*** ** = 14)**	**Ritlecitinib 10 mg (** ***n*** ** = 17)**	**Ritlecitinib 30 mg (** ***n*** ** = 34)**	**Ritlecitinib 50 mg (** ***n*** ** = 43)**	**Ritlecitinib 200/30 mg (** ***n*** ** = 28)**	**Ritlecitinib 200/50 mg (** ***n*** ** = 33)**
Overall study period (up to week 48 and including follow‐up)							
Patients with AEs	14 (82.4)	13 (92.9)	12 (70.6)	27 (79.4)	38 (88.4)	25 (89.3)	29 (87.9)
Most common AEs occurring in ≥15% of patients in any treatment group							
Nasopharyngitis	0	3 (21.4)	3 (17.6)	4 (11.8)	5 (11.6)	8 (28.6)	4 (12.1)
Folliculitis	1 (5.9)	1 (7.1)	3 (17.6)	3 (8.8)	5 (11.6)	8 (28.6)	9 (27.3)
Upper respiratory tract infection	2 (11.8)	3 (21.4)	0	7 (20.6)	6 (14.0)	2 (7.1)	10 (30.3)
Urticaria	3 (17.6)	1 (7.1)	0	4 (11.8)	5 (11.6)	3 (10.7)	5 (15.2)
Headache	1 (5.9)	2 (14.3)	0	2 (5.9)	5 (11.6)	2 (7.1)	6 (18.2)
Permanent discontinuation from study or study drug due to AEs	3 (17.6)	0	1 (5.9)	2 (5.9)	2 (4.7)	1 (3.6)	2 (6.1)
Patients with SAEs	1 (5.9)^a^	0	0	0	0	1 (3.6)^b^	1 (3.0)^a^
Patients with severe AEs	1 (5.9)	0	1 (5.9)	0	0	0	1 (3.0)

Abbreviations: AE, adverse event, SAE, serious adverse event.

^a^ Abortion spontaneous; unrelated to the study treatment.

^b^ Suicidal behavior and chemical poisoning; unrelated to the study treatment.

The incidence of serious AEs (SAEs), severe AEs, and permanent and temporary discontinuations across all treatment groups was not dose dependent. SAEs were reported in three patients: one patient in the 200/50‐mg group (abortion spontaneous), one patient in the 200/30‐mg group (suicidal behavior and chemical poisoning), and one patient in the placebo to 50‐mg group (abortion spontaneous). All SAEs were considered by the investigator as unrelated to study treatment. Eleven patients permanently discontinued the study due to AEs (≤3 patients per treatment group). The most frequently reported AEs that led to permanent discontinuation were pregnancy (three patients) and urticaria (two patients). No deaths, serious or opportunistic infections, major adverse cardiovascular events (MACE), thromboembolic events, malignancies, or nonmelanoma skin cancer were reported. Two events (1.1%) of HZ were reported in the 200/50‐ and 50‐mg groups; neither led to treatment discontinuation. Herpes simplex was reported in five patients (2.7%, reported in ≤2 patients in any treatment group), all events of which were mild in severity and resolved. Eight patients experienced neurological AEs that were equally distributed across treatment groups. Three patients (one each in the ritlecitinib 200/50‐, 50‐, and 30‐mg treatment groups) experienced sensorineural hearing loss. Audiological events were identified from protocol‐specified audiological testing; none were spontaneously reported. No events met criteria for central hearing disorder.

Small, early decreases from baseline were observed in hemoglobin, platelet, and neutrophil levels with ritlecitinib; however, levels remained generally stable up to week 48. Early and transient decreases from baseline in absolute lymphocytes were also observed, which appeared to be dose dependent and were more pronounced in patients who received the 200‐mg loading dose. No patients met the discontinuation criteria for changes in hemoglobin, platelet, neutrophil, or lymphocyte levels. A grade 3 decrease in neutrophil count was reported in one patient in the ritlecitinib 200/50‐mg group (Supporting Information Table S2). One patient in the ritlecitinib 200/50‐mg group had a grade 3 decrease in lymphocyte count (most abnormal value 0.46 × 10^9^/L on day 281). This patient had experienced an event of upper respiratory tract infection on study days 268–276. Creatine kinase levels >2 × upper limit of normal (ULN) occurred in 17 patients in the ritlecitinib groups and in two patients in the placebo to ritlecitinib 50‐mg group; no cases of rhabdomyolysis were reported (Supporting Information Table S3). Small, transient increases in low‐density lipoprotein cholesterol (>1.2 × ULN) were observed in patients who received the 200‐mg loading dose; no long‐term changes in lipid levels across the groups up to week 48 were reported. The incidence of elevations in hepatic enzymes was low and not dose dependent; two patients in the placebo to ritlecitinib 50‐mg group had increases in alanine aminotransferase (ALT) of >3 × ULN and three patients in the ritlecitinib groups (10 mg and higher) had aspartate aminotransferase (AST) increases >3 × ULN. There were no potential Hy's law cases, defined as cases in which hepatocellular drug‐induced liver injury with jaundice indicates a serious reaction.^45^ No patients met the discontinuation criteria for changes in ALT, AST, or creatine kinase levels.

## 4 DISCUSSION

This subgroup analysis evaluated the efficacy and safety of Asian patients from the ALLEGRO phase 2b/3 study. Overall, clinician‐ and patient‐reported efficacy outcomes in the Asian subpopulation were consistent with those of the overall study population and with previous studies.^44^, ^46^ Ritlecitinib demonstrated clinical efficacy in the Asian subpopulation. Greater SALT ≤20 and SALT ≤10 responses were observed with ritlecitinib ≥30 mg versus placebo at week 24, with continued improvement through week 48. At week 24, 9%–36% and 6%–27% of patients treated with ritlecitinib doses ≥30 mg achieved SALT score ≤20 and SALT score ≤10, respectively. These results are consistent with the overall study population, where 14%–31% and 11%–22% of patients achieved SALT score ≤20 and ≤10, respectively, at week 24.^44^

Improvement up to week 48 in EBA and ELA was also observed across all ritlecitinib treatment groups. Additionally, higher rates of patient‐reported improvement in AA were observed with ritlecitinib treatment versus placebo at week 24.

In the total study population, efficacy was maintained or continued to improve through week 48^44^; based on the data presented here, a similar effect was observed in Asian patients. Overall, clinician‐ and patient‐reported efficacy outcomes in Asian patients were consistent with those in the total study population.^44^

A subanalysis of the adolescent subset of patients was previously conducted in the ALLEGRO phase 2b/3 study and showed that the efficacy results in adolescents with AA were consistent with those in the overall study population.^47^ As the current subanalysis of the Asian subpopulation showed results consistent with the overall study population, it is expected that the efficacy of ritlecitinib in Asian adolescent patients would also be consistent with the overall study population.

Ritlecitinib had an acceptable safety profile and was well tolerated at all doses in the Asian subpopulation up to week 48, and no new or distinct safety signals were observed compared with the overall study population.^44^ No dose‐dependent trends in SAEs, severe AEs, AEs leading to discontinuation/dose reduction, or other significant AEs were observed in the Asian subpopulation. No deaths, malignancies, MACE, pulmonary embolisms, or opportunistic infections were reported in Asian patients. Two patients had AEs of HZ that were mild in severity. Audiological evaluation did not reveal any central hearing disorder from ritlecitinib, and no serious neurological AEs were reported. Dose regimens with a 200‐mg loading dose had a higher incidence of some AEs, such as folliculitis and dizziness, compared with the respective dose groups (50 mg or 30 mg) without a loading dose, and larger decreases in some hematological parameters such as lymphocytes. Overall, the safety profile suggests that there are no risks unique to Asian patients.

This subgroup analysis was limited by the small numbers of patients in each treatment group and therefore post hoc tests for statistical significance were not performed.

In conclusion, treatment with ritlecitinib demonstrated clinical efficacy and an acceptable safety profile over 48 weeks in Asian patients with AA and ≥50% hair loss, which was consistent with the overall population from the ALLEGRO‐2b/3 study.

## FUNDING INFORMATION

This study was sponsored by Pfizer Inc.

## CONFLICT OF INTEREST STATEMENT

X.Z. is a clinical trial investigator for Pfizer. Y.Y. and W.S. declare no conflicts of interest. Y. Sheng is a clinical trial investigator for Pfizer, Eli Lilly, AbbVie, Reistone Biopharma, Sanofi, Sichuan Kelun Pharmaceutical, and Suzhou Zelgen Biopharmaceuticals. M.K. is a clinical trial investigator for AbbVie, Bristol Myers Squibb, Eli Lilly Japan Inc., and Pfizer. O.K. is a clinical trial investigator for Eli Lilly Japan Inc. and Pfizer. C‐C.L. is a clinical investigator for Pfizer, Eli Lilly, AbbVie, Teva, Cerner Enviza, and Janssen. G.S., R.W., S.H., Q.S., Y. Shen, and M.S‐Y. are employees of and hold stock or stock options in Pfizer Inc. Taisuke Ito is a clinical trial investigator for Eli Lilly Japan I and Pfizer, and is an Editorial Board member of *Journal of Dermatology* and a co‐author of this article. To minimize bias, he was excluded from all editorial decision‐making related to the acceptance of this article for publication.

## ETHICS STATEMENT

The protocol was reviewed and approved by the institutional review boards or ethics committees of the participating institutions. The study was conducted in accordance with the International Ethical Guidelines for Biomedical Research Involving Human Subjects (Council for International Organizations of Medical Sciences 2002), ICH Guideline for Good Clinical Practice, and the Declaration of Helsinki. Written informed consent was obtained from each patient, parent, or patient's legal representative.

## TRIAL REGISTRIES

ClinicalTrials.gov: NCT3732807.

## Supporting information


Data S1.

## Data Availability

Upon request, and subject to review, Pfizer will provide the data that support the findings of this study. Subject to certain criteria, conditions, and exceptions, Pfizer may also provide access to the related individual de‐identified participant data. See https://www.pfizer.com/science/clinical‐trials/trial‐data‐and‐results for more information.
