# Supplementary material for: Efficacy and safety of ritlecitinib in Asian patients with alopecia areata: A subgroup analysis of the ALLEGRO phase 2b/3 trial
Source: J Dermatol. 2025 Mar 12;52(4):603–14. doi: 10.1111/1346-8138.17539 (PMC11975179; doi:10.1111/1346-8138.17539)
Supplement: Supplementary file 1 — Data S1. [file JDE-52-603-s001.zip › 1015_Asia_subgroup_supplemental_04SEP2024.docx]

**Efficacy and safety of ritlecitinib in Asian patients with alopecia areata: a subgroup analysis of the ALLEGRO phase 2b/3 trial**

**Supplemental Material**

**Table S1.** Overall disposition events summary up to Week 48.

| **n (%)** | **Placebo 🡪**  **Ritlecitinib**  **50 mg**  **(n=17)** | **Placebo 🡪 Ritlecitinib**  **200/50 mg**  **(n=14)** | **Ritlecitinib**  **10 mg**  **(n=17)** | **Ritlecitinib**  **30 mg**  **(n=34)** | **Ritlecitinib**  **50 mg**  **(n=43)** | **Ritlecitinib 200/30 mg**  **(n=28)** | **Ritlecitinib**  **200/50 mg**  **(n=33)** |
| --- | --- | --- | --- | --- | --- | --- | --- |
| Discontinued | 3 (17.6) | 1 (7.1) | 2 (11.8) | 4 (11.8) | 4 (9.3) | 3 (10.7) | 3 (9.1) |
| Adverse event | 2 (11.8) | 0 | 1 (5.9) | 1 (2.9) | 2 (4.7) | 1 (3.6) | 1 (3.0) |
| Lack of efficacy | 0 | 0 | 1 (5.9) | 1 (2.9) | 0 | 0 | 0 |
| Lost to follow-up | 0 | 0 | 0 | 1 (2.9) | 0 | 0 | 0 |
| Pregnancy | 1 (5.9) | 0 | 0 | 1 (2.9) | 0 | 0 | 1 (3.0) |
| Withdrawal by patients | 0 | 1 (7.1) | 0 | 0 | 1 (2.3) | 0 | 1 (3.0) |
| Other | 0 | 0 | 0 | 0 | 1 (2.3) | 2 (7.1) | 0 |
| Completed | 14 (82.4) | 13 (92.9) | 15 (88.2) | 30 (88.2) | 39 (90.7) | 25 (89.3) | 30 (90.9) |

**Table S2**. Laboratory abnormalities and CTCAE grade 2 or higher decreases in neutrophil and lymphocyte counts up to Week 48.

| n (%) | **CTCAE Grade** | **Placebo 🡪**  **Ritlecitinib**  **50 mg**  **(n=17)** | **Placebo 🡪 Ritlecitinib**  **200/50 mg**  **(n=14)** | **Ritlecitinib**  **10 mg**  **(n=17)** | **Ritlecitinib**  **30 mg**  **(n=34)** | **Ritlecitinib**  **50 mg**  **(n=43)** | **Ritlecitinib 200/30 mg**  **(n=28)** | **Ritlecitinib**  **200/50 mg**  **(n=33)** |
| --- | --- | --- | --- | --- | --- | --- | --- | --- |
| Neutrophil count decreased | Grade 2^†^ | 0 | 1 (7.1) | 0 | 2 (5.9) | 1 (2.3) | 3 (10.7) | 2 (6.1) |
|  | Grade 3^‡^ | 0 | 0 | 0 | 0 | 0 | 0 | 1 (3.0) |
| Lymphocyte count decreased | Grade 2^§^ | 2 (11.8) | 1 (7.1) | 1 (5.9) | 1 (2.9) | 3 (7.0) | 4 (14.3) | 5 (15.2) |
|  | Grade 3^¶^ | 0 | 0 | 0 | 0 | 0 | 0 | 1 (3.0) |

Abbreviation: CTCAE, Common Terminology Criteria for Adverse Events.

^†^<1500-1000/mm^3^.

^‡^<1000-500/mm^3^.

^§^<800-500/mm^3^.

^¶^<500-200/mm^3^.

**Table S3.** Laboratory test abnormalities up to Week 48.

| **n (%)** | **Placebo 🡪**  **Ritlecitinib**  **50 mg**  **(n=17)** | **Placebo 🡪 Ritlecitinib**  **200/50 mg**  **(n=14)** | **Ritlecitinib**  **10 mg**  **(n=17)** | **Ritlecitinib**  **30 mg**  **(n=34)** | **Ritlecitinib**  **50 mg**  **(n=43)** | **Ritlecitinib 200/30 mg**  **(n=28)** | **Ritlecitinib**  **200/50 mg**  **(n=33)** |
| --- | --- | --- | --- | --- | --- | --- | --- |
| **Laboratory test abnormalities, n/N^†^ (%)** | | | | | | | |
| AST >3 × ULN | 0/17 | 0/14 | 1/17 (5.9) | 0/34 | 1/42 (2.4) | 1/28 (3.6) | 0/33 |
| ALT >3 × ULN | 2/17 (11.8) | 0/14 | 0/17 | 0/34 | 0/42 | 0/28 | 0/33 |
| CK >2.0 × ULN | 2/17 (11.8) | 0/14 | 1/17 (5.9) | 2/34 (5.9) | 5/42 (11.9) | 3/28 (10.7) | 6/33 (18.2) |
| LDL cholesterol (mg/dL) >1.2 × ULN | 0/0 | 0/3 | 1/2 (50.0) | 0/2 | 0/2 | 0/1 | 0/0 |
| Triglycerides (mg/dL) >1.3 × ULN | 0/17 | 0/14 | 2/17 (11.8) | 3/34 (8.8) | 2/42 (4.8) | 2/28 (7.1) | 2/33 (6.1) |

Abbreviations: ALT, alanine aminotransferase; AST, aspartate aminotransferase; CK, creatine kinase; ULN, upper limit of normal.

^†^n is number of participants with laboratory test abnormality; N is the total number of participants with at least one observation of the given laboratory test while on study period.
